# The effects of different types of dance interventions on the autonomic nervous system of college students with depressed mood

**DOI:** 10.3389/fpsyg.2025.1625090

**Published:** 2025-07-21

**Authors:** Ruixue Zhao, Jiayi Li, Jiameng Wang

**Affiliations:** ^1^Lu Xun Art College, Yan'an University, Yan'an City, Shanxi, China; ^2^Faculty of Physical Education, Yan'an University, Yan'an City, Shanxi, China

**Keywords:** dance movement therapy, depressed mood, autonomic nervous system, heart rate variability, self-rating depression scale

## Abstract

**Purpose:**

This study aims to compare the regulatory effects of different dance styles (such as modern dance, Chinese classical dance, jazz dance, and ballet) on depressive mood, with a particular focus on their impact on the autonomic nervous system function (e.g., HRV indicators). The goal is to identify the most therapeutically promising form of dance intervention and provide more effective supplementary approaches for individuals experiencing depressive mood.

**Methods:**

This study involved screening 1,500 university students for Depressive mood out of a total of 10,784 students at the university (Anonymize) (*p* > 0.05). From the 1,500 individuals identified with depressive mood, 150 were randomly selected for the intervention. These participants were then randomly assigned to five groups: group A (classical dance), group B (ballet), group C (jazz dance), group D (modern dance), and group E (control group). The intervention consisted of dance practice four times weekly for 20 min per session over 12 weeks. Baseline demographic data, including age, gender, height, weight, and Body Mass Index (BMI), were collected, and participants completed the Self-Rating Depression Scale (SDS) test before the intervention began. Data organization was performed using Excel, and SPSS 22.0 was used for analysis.

**Results:**

Before the intervention, Heart Rate Variability (HRV) indicators showed no significant differences between groups (*p* > 0.05). However, during the recovery period, heart rate (HR) was notably higher after modern dance than after the other dance forms. The HRV index was significantly higher than those in the ballet and classical dance groups (*p* < 0.05). The modern dance group also showed a significant reduction in the SDS index compared to the classical, ballet, and jazz dance groups (*p* < 0.05).

**Conclusion:**

Modern dance may, therefore, be more effective in promoting recovery for individuals with depressive mood than Chinese classical, ballet, or jazz dance.

## 1 Introduction

Understanding the complex relationship that exists between depressive mood and physiological regulation is crucial in addressing the growing mental health challenges among university students. Therefore, this study addresses various areas that are affected by this rising menace to society, each designed to build a comprehensive foundation for the current study. First of all, we outline the global burden of depression and highlight the growing prevalence of mental health issues within college populations. Next, we introduce Heart Rate Variability (HRV) as a valuable physiological biomarker for assessing autonomic nervous system function in individuals with depressive symptoms. Thirdly, we explore the therapeutic potential of movement-based interventions—specifically Dance Movement Therapy (DMT)—in improving emotional regulation and psychological well-being. Fourthly, we examine how different dance styles may variably influence HRV and mood outcomes based on their physical and expressive characteristics. Thereafter, we present the purpose and significance of the study, emphasizing the need to identify evidence-based, non-pharmacological interventions tailored to the needs of students experiencing depressive mood. Together, these fusions of problems and solutions provide the conceptual and empirical rationale for investigating dance-based interventions as a pathway to enhancing mental health and physiological resilience.

### 1.1 Addressing the global mental health crisis among college students

Depression now affects more than 300 million people worldwide, ranking as the most widespread mental illness and the leading cause of global disability (Karkou et al., 2019; Hellem et al., 2020). Women face a disproportionate burden, being 1.5 to 3 times more likely than men to experience depressive episodes (Hellem et al., 2020). Beyond emotional distress, depression impairs sleep, disrupts appetite, and compromises daily functioning, ultimately diminishing individuals’ quality of life and burdening families and communities alike (Schrier et al., 2013).

University students face rising mental health challenges, with depressive symptoms becoming increasingly common. Academic pressure, uncertainty about career prospects, financial strain, and personality-related vulnerabilities all contribute to this growing crisis (Kovess-Masfety et al., 2016; Viseu et al., 2018). Despite the availability of psychological services on many campuses, a substantial proportion of students remain undiagnosed or untreated (Gonzálvez et al., 2018). Nearly one in four reports experiencing symptoms such as anxiety, obsessive-compulsive behaviors, and interpersonal conflict (Wu et al., 2015). These patterns highlight the urgent need to implement preventive and therapeutic strategies that resonate with the specific needs of student populations (Beiter et al., 2015).

### 1.2 Heart rate variability (HRV) as a biomarker/physiological Indicator of depression

Several researchers have increasingly relied on Heart Rate Variability (HRV) as a robust, non-invasive biomarker of autonomic nervous system (ANS) function. HRV reflects the interplay between sympathetic and parasympathetic activity and reveals how the body responds to internal and external stressors (Mitsuishi, 2022). Individuals diagnosed with depression, anxiety, or panic disorders often exhibit lower resting HRV, indicating impaired vagal tone and diminished emotional regulation (Niemela et al., 2008; Hillman et al., 2009).

Lower HRV correlates with heightened emotional reactivity, persistent rumination, and maladaptive stress responses—all common features of depressive states (Koch, 2007; Dell’Acqua et al., 2020; Hamilton and Alloy, 2017). People with low HRV frequently struggle to regulate negative affect, placing them at greater risk of developing or intensifying depressive symptoms (Hartmann et al., 2019; Messerotti et al., 2015). These findings position HRV not only as a diagnostic marker but also as a promising target for intervention (Carnevali et al., 2018; Park et al., 2014).

### 1.3 Dance as a movement-based intervention for depression

As concerns grow over the side effects and limited efficacy of pharmacological treatments, researchers and clinicians have turned toward alternative interventions. Among these, DMT has emerged as a promising, low-risk method for improving emotional well-being (Punkanen et al., 2017; Mala et al., 2012; Meekums et al., 2017).

DMT integrates physical activity with expressive art, harnessing the therapeutic power of movement, rhythm, posture, and interpersonal connection (3rd European Association of Dance Movement Therapy (EADMT) Conference, 2018). Through structured and improvised motion, dance facilitates emotional release, enhances self-awareness, and promotes social bonding—benefits particularly vital for individuals who struggle with verbal therapy or emotional articulation (Karkou et al., 2019; Hellem et al., 2020). The dual impact of dance on the body and psyche renders it especially suitable for mental health interventions.

### 1.4 Dance styles and their unique impact on HRV and emotional recovery

Not all dance styles engage the body and emotions in the same way. Variations in rhythm, expressiveness, and physical demand can produce differing effects on the ANS. For instance, modern dance, which encourages free-form movement and emotional expression, has shown a stronger association with improvements in HRV metrics—particularly the standard deviation of normal-to-normal intervals (SDNN)—a key indicator of autonomic adaptability (Varas-Díaz et al., 2020; Gruzelier et al., 2014).

In contrast, although ballet and jazz dance offer both aesthetic and physical engagement, they appear to yield more modest gains in HRV modulation. Studies have consistently shown that control groups with no movement-based interventions tend to exhibit the weakest autonomic recovery and lowest HRV levels (Hirahara et al., 2024). These findings suggest that the open, expressive format of modern dance may uniquely support emotional regulation and offer a superior therapeutic effect for individuals with depressive symptoms (Koch et al., 2019; Salihu et al., 2021).

### 1.5 Objectives and contribution of the present study

This study explores how different dance styles affect HRV and mood regulation in female college students experiencing depressive mood. By evaluating neuroelectrophysiological changes in ANS function, the research aims to determine which dance interventions most effectively promote physiological resilience and emotional well-being.

The findings aim to inform the design of targeted, evidence-based dance therapy programs, offering an alternative or complement to conventional treatments for depression. By tailoring interventions to the unique physiological and psychological responses associated with each dance form, this study may help expand mental health care options for vulnerable student populations and beyond.

## 2 Methods

### 2.1 Subjects

This research screened 1,500 students with depressive mood from a total of 10,784 university students at the university (Anonymize) (*p* > 0.05). From these 1,500 individual samples, 150 students with depressive mood were randomly selected as intervention subjects. All the participants were recruited before April 1, 2023. Eligible participants were expected to meet the following inclusion criteria: (1) registration with the university’s student management center before April 1, 2023, and (2) voluntary participation after receiving comprehensive information about the study and providing written informed consent. More so, the exclusion criteria included a history of hypertension, obesity, cardiovascular diseases, metabolic disorders, smoking, or consumption of stimulants within 12 h prior to the study. The Ethics Committee of the university approved this study. All participants provided informed consent.

### 2.2 Screening procedure and diagnostic criteria for depressive mood

We administered the Self-Rating Depression Scale (SDS) (Zung, 1965) to all 150 participants. Professional psychologists interpreted the SDS results to ensure diagnostic reliability. The SDS includes 20 items, each scored on a 4-point Likert scale: 1 (“Never”), 2 (“Sometimes”), 3 (“Frequently”), and 4 (“Always”). Items 2, 5, 6, 11, 12, 14, 16, 17, 18, and 20 are reverse-coded. We calculated the total raw score and then multiplied it by 1.25 to obtain the standard score, rounding to the nearest whole number. According to Chinese normative data, we classified standard scores as follows: (1) Mild depression: 53–62; (2) Moderate depression: 63–72; (3) Severe depression: >72. Each participant underwent an individual interview with a psychology expert to assess their depressive mood. Subsequently, all 150 participants were diagnosed with mild to severe depressive mood (with depression indices ranging from 58 to 70) and included as experimental subjects. A one-way ANOVA was conducted to confirm that there were no statistically significant differences among the groups across various indicators (*p* > 0.05), ensuring group homogeneity. The final valid sample sizes were as follows: Group A (Classical Dance) consisted of 29 participants; Group B (Ballet) consisted of 30 participants; Group C (Jazz Dance) consisted of 28 participants; Group D (Modern Dance) consisted of 27 participants, and; Group E (Control) consisted of 28 participants. Each intervention group trained under the supervision of a certified dance instructor in a professional dance studio. To ensure protocol fidelity, we monitored all sessions via video recordings. The intervention lasted 12 weeks, during which participants practiced dance four times per week for 20 min per session. Table 1 outlines the detailed intervention plans and summarizes the defining features of each dance style assigned to the groups below.

**Table 1:** Physical morphological characteristics and depression index of 29 experimental subjects (*x–* ± *s*).

Gender (*N* = 29)	Age (yrs)	Height (cm)	Weight (kg)	BMI (kg/m^2^)	SDS index
A (29)	18.76 ± 0.57	164.10 ± 2.86	51.03 ± 4.35	19.73 ± 1.73	58.31 ± 6.70
B (30)	18.53 ± 0.43	165.23 ± 3.96	52.53 ± 3.89	19.29 ± 1.69	60.10 ± 6.13
C (28)	19.28 ± 0.59	163.56 ± 2.89	54.78 ± 3.87	20.61 ± 1.73	57.67 ± 10.13
D (27)	18.94 ± 0.33	163.50 ± 2.86	52.13 ± 4.98	19.62 ± 1.83	58.58 ± 7.20
E (28)	20.03 ± 2.34	163.16 ± 1.47	52.22 ± 4.43	19.96 ± 1.26	56.36 ± 8.84

A denotes classical dance; B ballet; C jazz; D modern dance; E control group.

### 2.3 Basic information

The following are the detailed intervention plans for each group and the dance characteristics of the dances practiced by each group.

### 2.4 Group A: Chinese classical dance (29 participants)

*“The Brocade”*—Chinese classical dance is characterized by elegant, refined movements, emphasizing body fluidity and emotional expression. It has a positive regulatory effect on the autonomic nervous system, mainly by enhancing body awareness, relaxation, emotional regulation, and stress relief (Xiao-Qi et al., 2023; Lanye, 2022).

### 2.5 Group B: ballet (30 participants)

The ballet variation from *“Sleeping Beauty”* features graceful, structured movements and technical precision, showcasing character and the noble temperament of classical ballet. Long-term ballet training can enhance parasympathetic nervous system activity. Studies have shown that after 17 weeks of ballet training, young female dancers exhibited significantly improved parasympathetic indicators, indicating enhanced ANS regulation with no adverse health effects (Nakamura et al., 2015).

### 2.6 Group C: jazz dance (28 participants)

The jazz dance routine *“Nice to Meet You”* combined with Doja Cat’s *“Boss B—tch”* features strong rhythm, confident expressiveness, and flexible body movements, highlighting individuality and dynamism. Expectedly, jazz dance training can improve heart rate variability and ANS function, alleviate anxiety and depression, and promote psychological well-being (Hulbert et al., 2025; Martins et al., 2024).

### 2.7 Group D: modern dance (27 participants)

*“Ripples”* is a modern dance piece characterized by fluidity and layered movement, emphasizing body extension, energy transmission, and natural emotional expression. Modern dance training and performance induce dynamic changes in sympathetic (arousal) and parasympathetic (relaxation) activity. During performance, heart rate increases and parasympathetic activity decreases, but returns to baseline quickly post-performance, indicating strong neuroregulatory capacity (Zaragoza et al., 2020; DiPasquale et al., 2018).

### 2.8 Group E: control group (28 participants)

No intervention was applied; participants continued their usual daily routines. The names, characteristics, choreography, BPM, and duration of the four dance types are shown in Table 2.

**Table 2:** Detailed information on 4 types of dances.

Dance name	Dance type	Choreography	Rhythm (bpM)	Duration
Variation of “Sleeping Beauty”	Ballet	Marius. Petipa	93	2 min
“The Brocade”	Classical Dance	Zhang Yue	120	2 min
“Ripples”	Modern Dance	Zhang Disha	79	2 min
Nice to meet you + Doja cat “Boss B-Tch”	Jazz	Ando AA, MIJU	140	2 min

#### 2.8.1 Dance characteristics

One dance major conducted a detailed Beat test, documenting key dance characteristics, as presented in Table 2. Additionally, baseline demographic data, including age, gender, height, weight, and body mass index (BMI), were collected. Participants completed the SDS test before initiating the dance intervention. In addition, ANS assessments were conducted before the intervention and at Weeks 3, 6, 9, and 12 during the intervention. On the first day after completing the 12-week dance intervention, participants underwent another SDS test to evaluate the effectiveness of different dance types on depressive mood.

#### 2.8.2 Body composition measurements

We assessed body composition for all participants before the intervention using the InBody 720 body composition analyzer (manufactured by Biopace Co. Ltd., Korea). This measurement served to control for physical health variability and ensure that body composition did not bias the interpretation of HRV or mood-related outcomes.

#### 2.8.3 HRV testing

Two medical professionals conducted HRV testing and explained the procedures and precautions to participants with depression. HRV indices were recorded using the UbpulseT1 system (manufactured by LAXTHA, Korea). To ensure consistency, HRV testing was conducted before the intervention and at Weeks 3, 6, 9, and 12 during the intervention. For each session, participants rested quietly in a dimly lit room for 20 min to achieve mental calmness. Resting heart rate (RHR) was checked to ensure it ranged between 60 and 100 beats per minute. Participants then sat upright for three HRV tests in a resting state, with the average of the three results used as the final measure. Detailed HRV metrics are summarized in Table 3.

**Table 3:** HRV index parameters.

HRV index	Definition	Parameter description
HR (beats/min)	Mean heart rate	Reflects the average level of the RR interval
SDNN (ms)	The standard deviation of all normal adjacent RR intervals	A simple metric for assessing autonomic activity
LF (ms^2^)	Low-frequency power	Reflects sympathetic activity
HF (ms^2^)	High-frequency power	Reflects parasympathetic activity
TP (ms^2^)	Total energy	Reflects the activity of the autonomic nervous system as a whole and can be used to assess the regulatory capacity of the autonomic nervous system
LFnorm (n.u.)	LF/(LF + HF)*100	Directly reflect changes in sympathetic regulation
HFnorm (n.u.)	HF/(LF + HF)*100	Directly reflects changes in parasympathetic neuromodulation
LF/HF	Low-frequency power/high-frequency power	Sympathetic and parasympathetic balance
HRV-index	HRV index	Variation in cycle-by-cycle HRV

#### 2.8.4 ANS testing

ANS testing was conducted simultaneously with HRV testing. The UbpulseT1 HRV system (LAXTHA Inc., South Korea) was used to measure both HRV and ANS data concurrently.

### 2.9 Experimental setting

To minimize external factors that could influence the results, the experimental environment and timing were standardized. The laboratory temperature was maintained at 22–24°C with a relative humidity of 40–60% RH. Experiments were conducted daily between 14:00 and 15:00. To avoid potential cardiovascular influences, participants refrained from consuming caffeine, alcohol, nicotine, or engaging in vigorous physical activities within 12 h prior to the experiments. During dance interventions, the music volume was set at 80 decibels. All experimental data were meticulously recorded.

### 2.10 Data entry and statistical analysis

Two researchers independently entered the HRV and SDS data and cross-checked for any missing entries. When less than one-third of a participant’s data was missing, we substituted the missing values with the average. However, if more than one-third of the data was absent, we excluded that participant’s data from further analysis. Besides, data organization was completed in Excel, while statistical analysis was performed using SPSS 22.0. We summarized general demographic information using frequencies and percentages, and expressed normally distributed quantitative data as means and standard deviations, expressed as *x–* ± *s*. To evaluate the impact of the four dance styles on ANS function in participants with depressive mood, we conducted a repeated one-way ANOVA. Statistical significance for all tests was set at *p* < 0.05.

## 3 Results

There is a linear relationship between SDS scores and HRV-related indicators (such as SDNN and LF/HF), with more severe depression correlating with more pronounced HRV abnormalities (Wang et al., 2013). Additionally, SDS scores show a significant negative correlation (*p* < 0.05) with the very low-frequency (VLF) component of HRV (*ρ* = −0.70, *p* < 0.05) (Kono et al., 2022). This indicates a certain correlation between HRV data and SDS scale results, particularly concerning parameters such as SDNN, LF/HF, and VLF, which reflect the physiological basis of depressive mood. Therefore, the results of this study primarily focus on the analysis of HRV data.

### 3.1 Differences in HRV indices of the experimental subjects before the dance intervention

Table 4 presents the HRV and SDS indices before the dance interventions, revealing no statistically significant differences among participants (*p* > 0.05).

**Table 4:** Differences in HRV indices before the intervention (*x–* ± *s*).

Variate	A (29)	B (30)	C (28)	D (27)	E (28)	*F*	*p*	*Post-hoc*
HR (bpm/min)	95.93 ± 11.69	93.79 ± 13.31	91.66 ± 13.74	92.76 ± 15.10	87.86 ± 9.34	1.547	0.192	NS
SDNN (ms)	55.93 ± 18.57	54.86 ± 18.74	53.62 ± 18.06	55.48 ± 20.36	65.96 ± 27.93	1.594	0.179	NS
HRV index	14.06 ± 4.44	12.88 ± 4.07	14.49 ± 6.72	13.89 ± 4.77	14.31 ± 5.07	0.518	0.671	NS
SDS index	58.31 ± 6.70	60.10 ± 6.13	57.67 ± 10.13	58.58 ± 7.20	56.37 ± 8.84	0.839	0.503	NS
LF (ms^2^)	7.05 ± 0.84	6.83 ± 1.01	7.15 ± 1.20	6.95 ± 0.98	6.90 ± 1.13	0.428	0.788	NS
HF (ms^2^)	6.01 ± 0.96	5.73 ± 1.14	6.17 ± 1.00	5.93 ± 1.01	6.22 ± 1.11	0.984	0.418	NS
LFnorm (n.u.)	54.14 ± 3.36	54.57 ± 3.00	53.58 ± 2.36	54.08 ± 2.48	54.15 ± 2.87	0.441	0.779	NS
HFnorm (n.u.)	45.86 ± 3.36	45.39 ± 2.98	46.37 ± 2.48	45.89 ± 2.83	47.31 ± 2.92	1.830	0.126	NS
LF/HF	1.19 ± 0.18	1.21 ± 0.15	1.16 ± 0.11	1.18 ± 0.12	1.12 ± 0.16	1.576	0.186	NS
TP (ms^2^)	7.89 ± 0.77	7.90 ± 0.96	8.01 ± 1.34	7.81 ± 1.05	7.80 ± 0.90	0.200	0.938	NS

A denotes classical dance; B ballet; C jazz; D modern dance; E control group. SDNN, the standard deviation of all normal adjacent RR intervals, a simple metric for assessing autonomic activity. NS, there is no statistical difference between each group. *p* < 0.05 indicates that this difference is unlikely to have occurred by chance.

**Figure 1:**
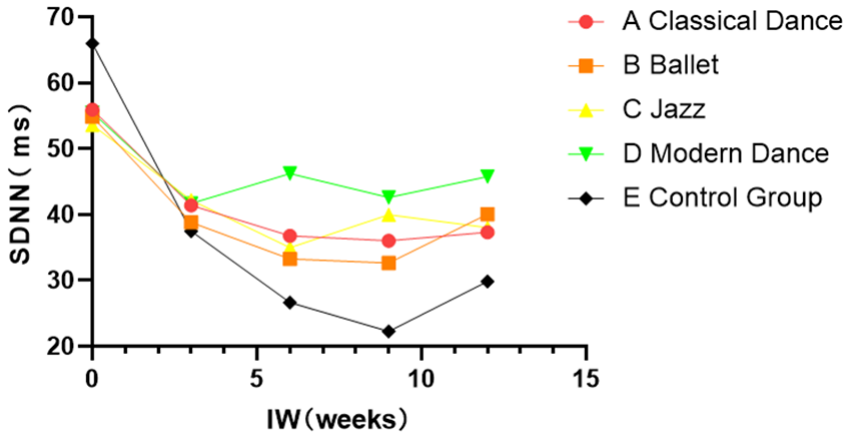
Change in SDNN during the recovery period (*p* < 0.05) indicates that this difference is unlikely to have occurred by chance.

**Figure 2:**
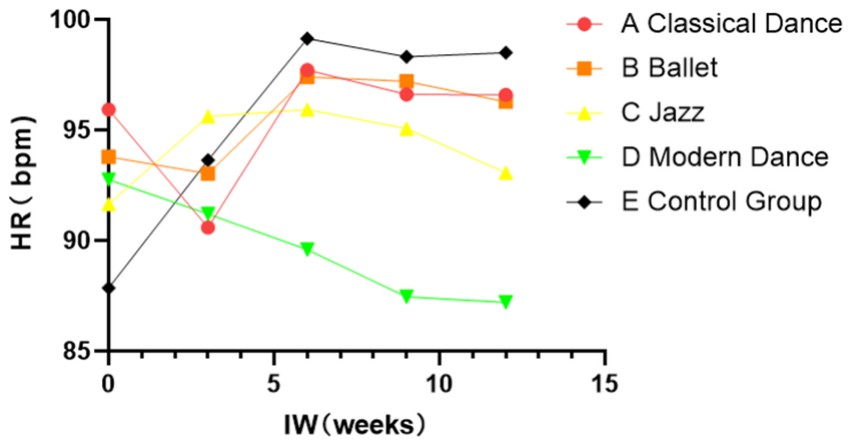
Change in HR during the recovery period (*p* < 0.05) indicates that this difference is unlikely to have occurred by chance.

That said, Table 5 demonstrates significant differences in heart rate HR and standard deviation of normal-to-normal intervals SDNN intervals during the recovery period across the different dance styles (*p* < 0.05). Moreover, classical, ballet, jazz dance, and the control group led to significantly higher recovery HR compared to modern dance (*p* < 0.05). Conversely, SDNN was notably lower after classical, ballet, jazz dance, and the control group than after modern dance (*p* < 0.05). This suggests that the modern dance group demonstrates stronger autonomic nervous system regulation and better overall health. Figures 1, 2 illustrate these patterns: over the 12-week dance intervention period, the control group exhibited significantly higher resting heart rates compared to the classical dance, ballet, jazz dance, and modern dance groups. The lowest RHR was observed in the Modern Dance group (*p* < 0.05). This suggests that modern dance may better activate the parasympathetic nervous system and enhance recovery mechanisms. The physiological effects of different dance styles should be considered separately, as modern dance may combine both physical challenges and psychological and emotional regulation. Intriguingly, SDNN values were highest post-modern dance, indicating greater autonomic recovery compared to the other dance styles and control group (*p* < 0.05).^1^

**Table 5:** Differential analysis of changes in indicators of time domain variability during different dance intervention processes (*x–* ± *s*).

Variate	IW	A (29)	B (30)	C (28)	D (27)	E (28)	*F*	*p*	*Post-hoc*
HR (bpm/min)	BI	95.93 ± 11.69	93.79 ± 13.31	91.66 ± 13.74	92.76 ± 15.10	87.86 ± 9.34	1.547	0.192	NS
	3	90.59 ± 11.56	93.03 ± 14.86	95.62 ± 14.19	91.21 ± 7.87	93.64 ± 8.87	4.864	0.001	A > D; B > D; C > D; E > D
	6	97.72 ± 11.81	97.41 ± 13.43	95.93 ± 11.39	89.59 ± 9.28	99.14 ± 6.84			
	9	96.62 ± 11.02	97.21 ± 8.95	95.07 ± 10.54	87.45 ± 8.74	98.32 ± 6.96			
	12	96.59 ± 10.73	96.28 ± 10.02	93.07 ± 11.42	87.21 ± 8.26	98.50 ± 9.32			
SDNN (ms)	BI	55.93 ± 18.57	54.86 ± 18.74	53.62 ± 18.06	55.48 ± 20.36	65.96 ± 27.93	1.594	0.179	NS
	3	41.41 ± 8.8	38.8 ± 8.71	42.45 ± 9.72	41.68 ± 10.10	37.46 ± 11.72	6.327	0.000	A < D; B < D; C < D; E < A, B, CD
	6	36.76 ± 14.53	33.28 ± 16.55	34.97 ± 13.06	46.21 ± 21.44	26.66 ± 12.27			
	9	36.03 ± 13.88	32.66 ± 13.14	39.97 ± 17.99	42.59 ± 19.50	22.25 ± 13.95			
	12	37.31 ± 18.50	40.07 ± 20.59	38.00 ± 17.16	45.76 ± 15.84	29.86 ± 6.93			

IW Intervention weeks; A denotes classical dance; B ballet; C jazz; D modern dance; E control group. BI, before intervention. *p* < 0.05 indicates that this difference is unlikely to have occurred by chance.

The analysis of frequency-domain variability indicators during different dance intervention processes revealed significant effects across various measures (Table 6). Different dance types had a significant impact on low-frequency (LF), high-frequency (HF), LFnorm, HFnorm, the LF/HF ratio, and the HRV index (*p* < 0.05). Specifically, the ballet group had significantly lower LF and HF values compared to the jazz and modern dance groups (*p* < 0.05). Additionally, the HF values in the jazz dance group (*p* < 0.05) were significantly higher than those in the classical dance group (*p* < 0.05). This implies that different dance forms have a significant impact on the ANS activity of the participants. Furthermore, the LF and HF values in the ballet group were both lower than those in the jazz and modern dance groups, suggesting that their effect on enhancing ANS regulation is relatively weaker. On the other hand, the HF value in the jazz dance group was higher than that in the classical dance group, indicating that jazz dance is more effective in activating the parasympathetic nervous system, which helps improve the balance between body and mind.

**Table 6:** Differences in frequency domain variation indices during different dance intervention processes (*x–* ± *s*).

Variate	IW	A (29)	B (30)	C (28)	D (27)	E (28)	F	*p*	*Post-hoc*
LF (ms^2^)	BI	7.05 ± 0.84	6.83 ± 1.01	7.15 ± 1.20	6.95 ± 0.98	6.90 ± 1.13	0.428	0.788	NS
	3	7.20 ± 0.67	6.83 ± 1.53	7.38 ± 0.92	7.28 ± 0.67	6.37 ± 1.62	16.418	0.000	B < C; B < D; A, B, C, D > E
	6	5.91 ± 0.98	5.49 ± 0.94	5.93 ± 0.82	6.18 ± 1.05	4.88 ± 1.86			
	9	6.12 ± 0.89	5.80 ± 1.05	6.39 ± 1.08	6.43 ± 1.21	4.35 ± 1.26			
	12	6.15 ± 1.02	6.12 ± 1.10	6.24 ± 1.09	6.33 ± 0.91	5.36 ± 1.01			
HF (ms^2^)	BI	6.01 ± 0.96	5.73 ± 1.14	6.17 ± 1.00	5.93 ± 1.01	6.22 ± 1.11	0.984	0.418	NS
	3	5.46 ± 1.04	5.52 ± 1.54	6.30 ± 0.89	6.27 ± 0.92	4.99 ± 1.34	8.231	0.000	B < C, D; C > A; A, B, C, D > E
	6	4.91 ± 1.06	4.45 ± 1.03	5.01 ± 0.95	5.31 ± 1.12	3.84 ± 1.68			
	9	4.84 ± 0.90	4.71 ± 1.07	5.11 ± 1.20	5.40 ± 1.09	3.76 ± 0.88			
	12	4.95 ± 1.15	4.97 ± 1.18	6.44 ± 0.67	5.48 ± 0.89	4.06 ± 0.92			
TP (ms^2^)	BI	7.89 ± 0.77	7.90 ± 0.96	8.01 ± 1.34	7.81 ± 1.05	7.80 ± 0.90	0.200	0.938	NS
	3	8.89 ± 0.92	8.24 ± 1.67	9.04 ± 0.93	11.73 ± 1.95	7.75 ± 1.73	3.304	0.013	C, D > E
	6	6.84 ± 0.93	6.63 ± 1.00	6.87 ± 0.77	7.10 ± 1.09	6.22 ± 1.56			
	9	6.80 ± 0.90	6.62 ± 0.92	7.12 ± 1.10	7.18 ± 1.07	5.57 ± 1.18			
	12	6.84 ± 1.01	7.01 ± 1.04	9.07 ± 1.18	7.38 ± 0.80	6.20 ± 1.15			
LFnorm (n.u.)	BI	54.14 ± 3.36	54.57 ± 3.00	53.58 ± 2.36	54.08 ± 2.48	54.15 ± 2.87	0.441	0.779	NS
	3	55.99 ± 1.74	56.27 ± 5.07	54.09 ± 2.59	53.89 ± 3.13	57.24 ± 3.64	1.949	0.014	D < A, B; C, D > E
	6	54.96 ± 5.43	55.56 ± 4.97	54.40 ± 4.02	53.97 ± 3.48	54.90 ± 8.08			
	9	55.88 ± 5.31	55.43 ± 5.43	55.89 ± 3.86	54.35 ± 3.20	55.59 ± 8.28			
	12	55.56 ± 3.96	55.36 ± 3.76	55.42 ± 3.77	53.65 ± 3.96	56.40 ± 6.09			
HFnorm (n.u.)	BI	45.86 ± 3.36	45.39 ± 2.98	46.37 ± 2.48	45.89 ± 2.83	47.31 ± 2.92	1.830	0.126	NS
	3	43.93 ± 1.82	43.73 ± 5.03	46.43 ± 3.36	46.12 ± 3.13	42.74 ± 3.64	2.024	0.013	D > A, B; D > E
	6	45.04 ± 5.43	44.49 ± 5.06	45.60 ± 4.02	46.03 ± 3.48	44.95 ± 8.00			
	9	44.12 ± 5.31	44.54 ± 5.53	44.11 ± 3.86	45.65 ± 3.20	44.41 ± 8.28			
	12	44.44 ± 3.96	44.48 ± 3.72	44.56 ± 3.75	46.35 ± 3.96	43.54 ± 6.08			
HRV-index	BI	14.06 ± 4.44	12.88 ± 4.07	14.49 ± 6.72	13.89 ± 4.77	14.31 ± 5.07	0.518	0.671	NS
	3	6.71 ± 2.98	7.39 ± 4.58	10.91 ± 7.05	11.28 ± 6.22	7.18 ± 1.38	14.112	0.000	D > A, B; A, B, C, D > E
	6	10.42 ± 3.40	9.02 ± 2.61	9.88 ± 3.87	12.81 ± 7.01	6.13 ± 1.78			
	9	10.57 ± 3.81	8.90 ± 2.63	10.53 ± 3.95	11.46 ± 4.41	6.06 ± 1.74			
	12	10.20 ± 3.53	10.64 ± 3.82	10.59 ± 3.75	12.66 ± 3.96	7.27 ± 2.39			
SDS index	BI	58.31 ± 6.70	60.10 ± 6.13	57.67 ± 10.13	58.58 ± 7.20	56.37 ± 8.84	0.839	0.503	NS
	12	56.19 ± 5.31	55.52 ± 5.76	55.59 ± 5.64	52.49 ± 5.96	56.91 ± 5.71	2.598	0.014	E > B, C, D; D < A, B, C

IW Intervention weeks; A denotes classical dance; B ballet; C jazz; D modern dance; E control group. BI, before intervention. *p* < 0.05 indicates that this difference is unlikely to have occurred by chance.

This may be related to the rhythm intensity, movement freedom, and emotional expression inherent in each dance style. In terms of normalized values, LFnorm was significantly lower in the modern dance group than in the ballet and classical dance groups (*p* < 0.05), while HFnorm was significantly higher in the modern dance group (*p* < 0.05). The suggestion that modern dance interventions help enhance parasympathetic nervous activity and reduce the relative dominance of the sympathetic nervous system indicates a regulatory shift of the autonomic nervous system toward relaxation and recovery. This may be related to the characteristics of modern dance, which emphasize free bodily expression and rhythmic variation, contributing to emotional soothing and the improvement of physical and mental balance. Relatedly, the HRV index was significantly higher than those in the ballet and classical dance groups (*p* < 0.05). This suggests that modern dance has a more positive effect on the regulation of the ANS. The free rhythm and dynamic movement patterns of modern dance may be more conducive to the activation of the parasympathetic nervous system, thereby increasing HRV and promoting relaxation and recovery. In contrast, ballet and classical dance, which are relatively static and emphasize posture maintenance, may result in lower HRV values. Congruently, participants in the modern dance group experienced a significantly greater reduction in the SDS scores compared to those in the classical, ballet, and jazz dance groups (*p* < 0.05).

#### 3.1.1 Analysis of within-group differences

To assess within-group changes, we conducted paired *t*-tests on SDS scores collected before and after the 12-week intervention. The results showed that all intervention groups, except for the Jazz group and the Control group, exhibited significant reductions in SDS scores after the intervention, indicating an improvement in depressive symptoms among the participants.

#### 3.1.2 Classical dance (the Brocade) group

The participants showed a significant reduction in depressive symptoms, with SDS scores decreasing from 58.31 ± 6.70 to 56.19 ± 5.31 (*t* = −2.34, *p* = 0.029).

#### 3.1.3 Ballet (variation of “Sleeping Beauty”) group

The pre-intervention SDS score declined from 60.10 ± 6.13 to 55.52 ± 5.76, also reflecting a significant improvement (*t* = −3.11, *p* = 0.008).

#### 3.1.4 Jazz (Nice to Meet You + Doja Cat’s “Boss B-Tch”) group

Although the pre-intervention SDS scores decreased from 57.67 ± 10.13 to 55.59 ± 5.64, this change was not statistically significant (*t* = −1.22, *p* = 0.241).

#### 3.1.5 Modern dance (Ripples) group

The participants in this group exhibited the most significant improvement, with SDS scores dropping from 58.58 ± 7.20 to 52.49 ± 5.96. Besides, the paired *t*-test results showed a significant decrease in SDS scores after the intervention (*t* = −4.52, *p* < 0.001).

#### 3.1.6 Control group

No meaningful change was observed in SDS scores, which slightly increased from 56.37 ± 8.84 to 56.91 ± 5.71. Also, the paired *t*-test results showed no significant change in SDS scores after the intervention (*t* = 0.56, *p* = 0.591).

#### 3.1.7 Between-group differences in depressive symptom reduction

To compare the effectiveness of the different dance interventions, we conducted a one-way analysis of variance (ANOVA) on the change in SDS scores across the five groups. The analysis revealed a significant difference among groups (*F* (4, 95) = 4.67, *p* = 0.002), indicating that the type of dance intervention influenced the degree of improvement in depressive symptoms. Further *post-hoc* analysis using Tukey’s HSD test further confirmed that: The modern dance group achieved the largest reduction in SDS scores, showing a statistically significant difference compared to the control group (*p* < 0.05).

Both the classical dance and ballet groups also demonstrated significant improvements, though to a lesser extent than the modern dance group. The jazz dance group did not differ significantly from the control group, suggesting a limited intervention effect. These findings highlight modern dance as the most effective style in reducing depressive mood, followed by classical dance and ballet, while jazz dance showed a comparatively weaker effect. The control group, which received no intervention, showed no notable change in depressive symptoms, reinforcing the importance of structured dance-based interventions.

**Figure 3:**
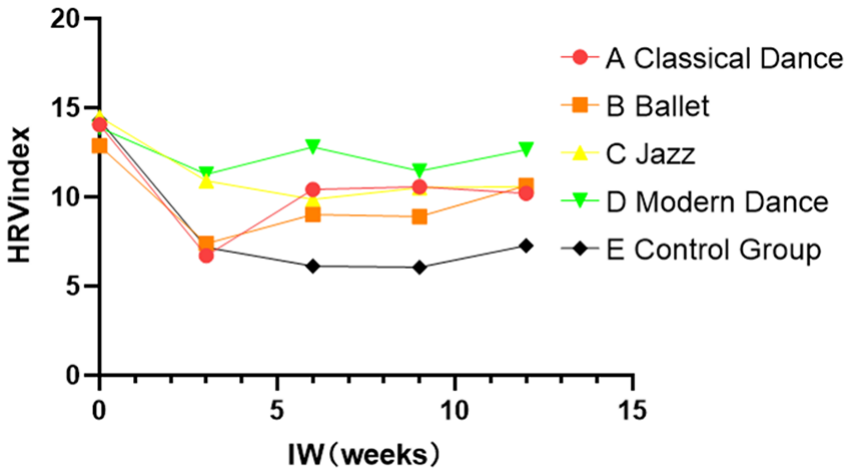
Change in HRV-index during the recovery period.

**Figure 4:**
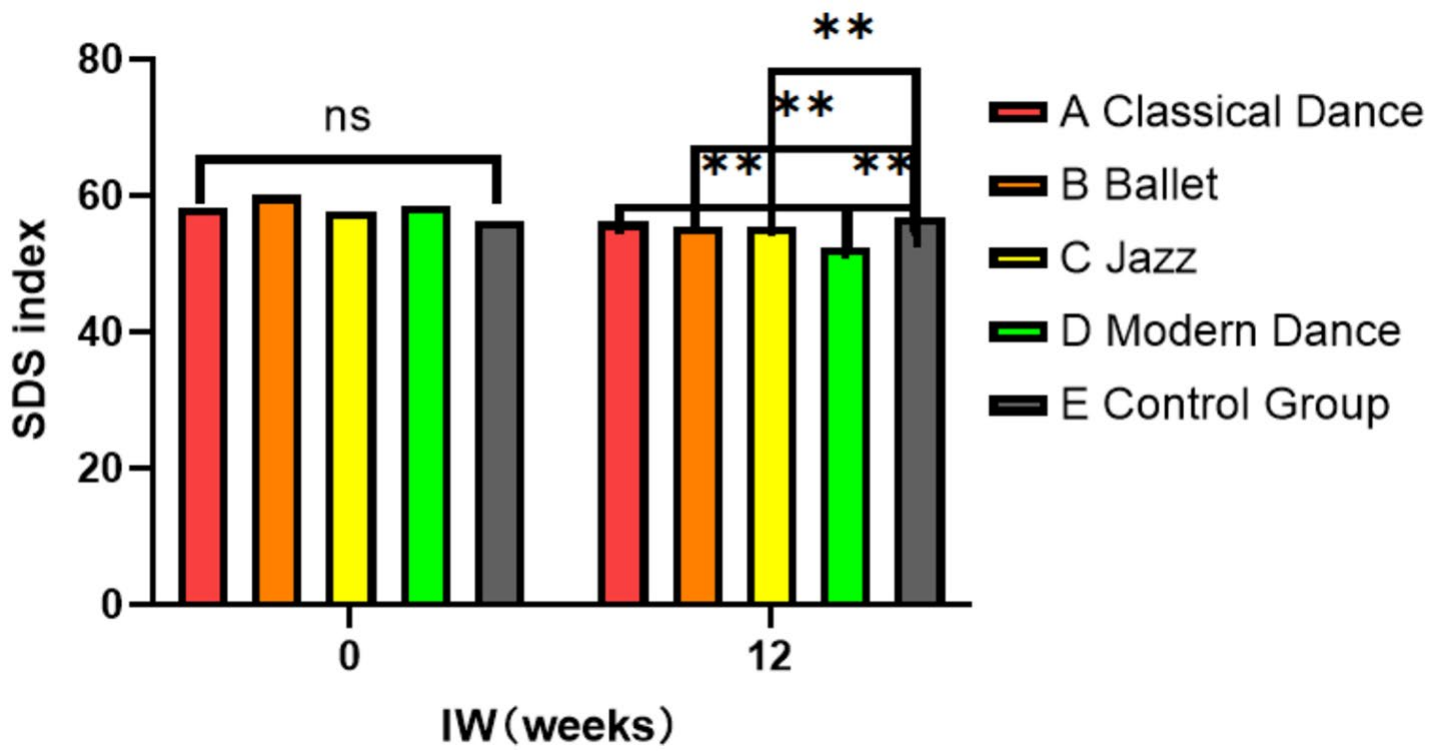
Change in HRV-index during the recovery period.

As illustrated in Figures 3, 4, the HRV index of the control group was significantly lower than that of the classical dance, ballet, jazz dance, and modern dance groups during the intervention. Among the dance intervention groups, the modern dance group demonstrated the most substantial improvement in HRV. All four dance intervention groups showed improvements in SDS scores compared to the control group. The modern dance group displayed the most notable improvement in depressive mood. An increase in HRV is associated with greater stress resilience and emotional regulation, suggesting that modern dance may be an effective intervention for helping students alleviate emotional distress.

## 4 Discussion

Heart Rate Variability (HRV) indicators in both the time and frequency domains have been linked to the severity of depression. For instance, Wang et al. (2013) found correlations between the Self-Rating Depression Scale scores and the time-domain index SDNN, as well as frequency-domain indicators. Similarly, large-sample studies from Taiwan and China demonstrated significant associations between frequency-domain indices and Hamilton Depression Scale scores (Ha et al., 2015; Chang et al., 2015), highlighting the potential diagnostic value of these indices in depression. Furthermore, time-domain indicators such as SDNN and RMSSD which are indicators of HRV reflecting the activity of the parasympathetic nervous system in the body, as well as pNN50 which is one of the indicators of heart rate variability, representing the percentage of the total number of normal heart rate intervals in an electrocardiogram that have a time difference greater than 50 ms between adjacent beats—tend to be lower in individuals with depressive mood compared to healthy controls (Liang, 2015). Previous research by Yang et al. also reported notable changes in these HRV indicators among individuals with depression who had never received medication (Ha et al., 2015; Chang et al., 2015; Liang, 2015). Intriguingly, SDNN values period after modern dance were significantly higher than those following classical, jazz, or ballet dance (*p* < 0.05). Moreover, we observed a slight upward trend in SDNN values after the modern dance intervention, suggesting a calming and restorative effect (Figure 1).

Previous studies have consistently shown that depressed patients often exhibit lower total power (TP) and HF components, along with higher LF/HF ratios (Chang et al., 2017). The most significant finding across these studies is the reduction in HF. Ha et al. (2015) found reduced TP and HF in elderly individuals with untreated depressive mood, while Shinba (2017) noted similar reductions in HF alongside elevated LF/HF in patients experiencing their first episode of depressive mood without medication. Similarly, Yeh et al. (2016) studied 618 individuals with depressive mood who had never taken or had not recently taken medication, finding decreased HF and increased LF/HF. Our research aligns with these findings in that the LFnorm value was significantly lower after modern dance compared to classical and ballet dances (*p* < 0.05). Additionally, HFnorm was significantly higher after modern dance (*p* < 0.05), and the LF/HF ratio was notably higher too. Besides, the HRV-index showed significantly higher values following modern dance than after classical, ballet dance, and the control group.

Numerous studies have examined how various dance forms, including ballet and modern dance, affect HRV in dancers, with differing results. Contrary to our findings, some research indicates that the HRV index for modern dance is significantly lower than for ballet (*p* < 0.05). Without mincing words, dance is both an art form and a form of exercise, with variations in style, movement intensity, and metabolism among ballet, classical, modern, and jazz dances. These differences influence the ANS in diverse ways (Kovess-Masfety et al., 2016), as varying intensities and rhythms in physical activity uniquely affect autonomic balance. For instance, aerobic exercises generally enhance autonomic control of cardiac function. In our study, modern dance, with a tempo of 79 beats per minute—the slowest of the four dance types—exhibited the most pronounced positive effect on the autonomic nervous system of patients with depressive mood. This suggests that the tempo of the dance could play a crucial role in regulating autonomic responses in individuals with depressive mood.

Our research indicates that modern dance is the most effective in regulating the autonomic nervous system of patients with depressive mood. However, several factors can influence the effectiveness of dance interventions, including individual differences, environmental conditions, temperature, diet, and lifestyle habits. Factors such as resting heart rate, respiration rate, and ambient noise during HRV measurements can also impact data collection accuracy (Kemp et al., 2010; Krypotos et al., 2011). Despite these limitations, our research demonstrates that modern dance leads to significant improvements in SDNN, LF/HF ratio, HRV index, and SDS index compared to classical dance, jazz dance, and ballet. Thus, modern dance appears to offer more substantial benefits for individuals with depressive mood.

## 5 Conclusion

This research highlights that modern dance interventions offer greater benefits for participants with depressive mood, particularly regarding standard deviation of normal-to-normal intervals, Heart Rate Variability, High Frequency, Low Frequency / High Frequency, and Overall Heart Rate Variability Index. In comparison, classical dance, jazz dance, and ballet yield less pronounced improvements. Therefore, when selecting dance interventions for addressing depressive mood, it is crucial to consider the tempo of the dance. Comparatively, modern dance may provide more significant advantages for the rehabilitation of patients with depressive mood compared to classical dance, ballet, and jazz dance.

Over and above, this study concludes that modern dance most effectively regulates the ANS in individuals with depressive mood, leading to greater improvements in HRV, HF, and the LF/HF ratio compared to classical dance, jazz dance, and ballet. Despite limitations such as small sample size and potential variability in measurement conditions, the findings suggest that modern dance’s unique tempo and intensity may enhance its therapeutic benefits for depressive mood. Therefore, incorporating modern dance into treatment plans could offer a more impactful approach to managing depressive mood and promoting overall mental health recovery. Taken together, modern dance, as an intervention method, may help enhance students’ emotional regulation and emotional resilience. For individuals struggling with depressive mood, it represents a promising option that mental health professionals may consider.

In conclusion, among the dance styles examined, modern dance emerged as the most effective in enhancing autonomic function and alleviating depressive symptoms. Its slower tempo and expressive form may enhance emotional resilience and parasympathetic activation, making it a valuable tool for mental health recovery. Mental health professionals may consider incorporating modern dance into intervention programs targeting depressive mood, particularly among university students. Nonetheless, this study faced limitations. A modest sample size and potential variability in testing conditions reduce the generalizability of the findings. Future research should aim to include more diverse populations, expand sample sizes, and control for individual and environmental confounders. Longitudinal studies examining the sustained psychological and physiological effects of different dance modalities will also help validate and extend these conclusions. Thus, modern dance represents a promising non-pharmacological intervention for depressive mood. Its capacity to improve HRV and emotional regulation supports its inclusion in personalized mental health strategies for young adults navigating psychological stress and affective disorders.

## Data Availability

The original contributions presented in the study are included in the article/supplementary material, further inquiries can be directed to the corresponding author.

## Footnotes

^1^*p* < 0.05 indicates that this difference is unlikely to have occurred by chance.
